# High incidence of interleukin 10 mRNA but not interleukin 2 mRNA detected in human breast tumours.

**DOI:** 10.1038/bjc.1997.311

**Published:** 1997

**Authors:** E. Venetsanakos, I. Beckman, J. Bradley, J. M. Skinner

**Affiliations:** Department of Histopathology, Flinders Medical Centre, South Australia.

## Abstract

**Images:**


British Journal of Cancer (1997) 75(12), 1826–1830
© 1997 Cancer Research Campaign

### High incidence of interleukin 10 mRNA but not interleukin 2 mRNA detected in human breast tumours

**E Venetsanakos[1], I Beckman[2], J Bradley[3] and JM Skinner[1]**

Departments of [1]Histopathology, [2]Microbiology and Infectious Diseases and [3]Clinical Immunology, Flinders Medical Centre, South Australia, Australia

**Summary** Despite the presence of a lymphocytic infiltrate in solid cancers, the failure for tumour growth to be contained suggests an inadequate immune response to the tumour. Poor cytotoxicity exerted by tumour-infiltrating lymphocytes (TILs) against tumour cells in vitro, combined with continued tumour growth in vivo, suggests deficiencies in TIL function or numbers. Various theories have been postulated to explain how tumour cells may escape immunosurveillance and control. One of the many hypotheses is the failure of production of cytokines, which are necessary for T cells to mediate their function. Thus, the expression of cytokine mRNA in human breast tumour sections was investigated by reverse transcriptase polymerase chain reaction (RT-PCR) with cytokine-specific primers. A relatively consistent finding was detection of interleukin (IL) 10 mRNA among the tumours. No IL-2 and little IL-4 mRNA was detected in the tumours. IL-6 and IL-10 mRNA was detected in only one and two of the normal breast tissues respectively. IL-2, IL-4 and tumour necrosis factor (TNF)-α mRNA was not detected in any of the normal breast tissues. The reduced function of TILs may be related to IL-10, which has known inhibitory effects on T-cell activation.

**Keywords:** tumour-infiltrating lymphocyte; breast tumour; cytokine

Within solid human tumours there is usually a mononuclear infiltrate consisting predominantly of T lymphocytes, with few B lymphocytes, natural killer (NK) cells and macrophages (Whiteside et al, 1986; Topalian and Rosenberg, 1990). The expression of HLA-DR, CD25 and CD71 (transferrin receptor), on the majority of tumour-infiltrating lymphocytes (TILs) from breast cancers suggested that the T cells have been activated in vivo (Whitford, et al, 1990; Chin et al, 1992; Whitford et al, 1992; Ostenstad et al, 1994). However, the failure to contain tumour growth suggests deficiencies in TIL function or numbers. In support of this, freshly isolated TILs show poor cytotoxicity against autologous and allogeneic tumour cells and poor proliferative responses to mitogen and alloantigen stimulation, although the cytotoxicity levels of TILs can be increased by stimulation with recombinant interleukin (rIL)-2 (Heo et al, 1987; Miescher et al, 1987; Reilly and Antognetti, 1991; Wimmenauer et al, 1991).

Selective expression of cytokines by TILs has been reported in melanoma, ovarian and brain tumours (Pisa et al, 1992; Merlo et al, 1993; Luscher et al, 1994). The local production of cytokines by TILs within the tumour microenvironment is crucial in mounting an immune response to tumour cells, and the presence of suppressive cytokines might thwart the effector response. Failure to stimulate the production of particular cytokines may inhibit the functions of TILs directed against the tumour cells. This study investigated the stable cytokine mRNA levels in freshly excised human breast tumours, using a reverse transcriptase polymerase chain reaction (RT-PCR) assay. The presence of cytokine mRNA was found to be heterogeneous among individual tumours with a

**Table 1** Clinical features of patients with breast cancer

| Case number | Age | Histology | Grade | LN involvement |
|---|---|---|---|---|
| I7 | 64 | IDC | III | 0/14 |
| I13 | 42 | IDC | III | ND |
| I16 | 38 | IDC | II | 1/11 |
| I18 | 63 | IDC | II | 2/18 |
| I19 | 80 | IDC | III | ND |
| I20 | 62 | IDC | II | 0 |
| I21 | 77 | ILC | ND | ND |
| I22 | 77 | IDC | III | 0/16 |
| I23 | 76 | IDC | II | 1/4 |
| I24 | 38 | ILC | ND | 0/12 |
| I25 | 68 | ILC | ND | ND |
| I26 | 56 | IDC | III | 0/35 |
| I27 | 78 | IDC | II | 0/20 |
| I28 | 55 | IDC | III | 2/20 |
| I30 | 33 | IDC | II | 0/14 |
| I31 | 58 | IDC | I | 0/11 |
| I48 | 61 | IDC | I | 0/15 |
| I62 | 57 | IDC | I | 0/16 |
| I64 | 48 | IDC | I | 12/19 |
| I66 | 57 | IDC | I | 2/23 |
| I70 | 45 | IDC | III | 2/25 |
| I72 | 69 | IDC | I | 1/20 |
| J17 | 70 | IDC | III | 2/26 |
| J18 | 41 | IDC | II | 4/15 |
| J24 | 74 | IDC | III | 2/15 |
| J33 | 42 | IDC | III | 32/38 |
| J38 | 66 | IDC | II | 0/18 |

Each breast tumour was characterized by histological examination and analysed independently. The above clinical features were recorded for this study, including the age of the patient, the type and grade (Bloom and Richardson) of the diagnosed breast tumour and the number of axillary lymph nodes with positive tumour involvement in the total number of lymph nodes examined histologically. IDC, infiltrating ductal carcinoma; ILC, invasive lobular carcinoma; LN, lymph node; ND, not determined.

*Received 13 May 1996*
*Revised 29 October 1996*
*Accepted 18 December 1996*

*Correspondence to:* E Venetsanakos, University Department of Medicine, QEII Medical Centre, 4th Floor, G Block, Verdun Street, Nedlands, WA 6009, Australia

**Table 2** Nucleotide sequences of primer pairs

| Gene | Primer sequence | | No. of base pairs of amplified product |
|---|---|---|---|
| β-Actin | Sense | TGACGGGGTCACCCACACTGTGCC | 661 |
| | Antisense | CTAGAAGCATTGCGGTGGACGATG | |
| IL-2 | Sense | ATGTACAGGATGCAACTCCTGTGTT | 458 |
| | Antisense | GTCAGTGTTGAGATGATGCTTTGAC | |
| IL-4 | Sense | GGGTCTCACCTCCCAACTGCT | 301 |
| | Antisense | CGAACACTTTGAATATTTCTCTCTC | |
| IL-6 | Sense | TGAACTCCTTCTCCACAAGCGC | 627 |
| | Antisense | GAAGACCCCTCAGGCTGGACT | |
| IL-10 | Sense | CTGAGAACCAAGACCCAGACATCA | 301 |
| | Antisense | CAATAAGGTTTCTCAAGGGGCTGG | |
| TNF-α | Sense | CGAGTGACAAGCCTGTAGCCC | 440 |
| | Antisense | TGATCCCAAAGTAGACCTGCCC | |
| CD3δ | Sense | GTACTGAGCATCATCTCGATG | 309 |
| | Antisense | CTGGACCTGGGAAAACGCATC | |

**Figure 1** Detection of amplified cytokine products in a representative breast tumour (I30) and normal breast tissue (Nb5). PHA-stimulated PBLs were used as positive controls for cytokine mRNA expression. Total RNA was isolated from frozen tissue sections, cDNA synthesized in a reverse-transcription reaction at 37°C and RT-PCR performed using cytokine-specific primers. Amplified products were analysed by gel electrophoresis. Amplified product sizes include: β-actin, 661 bp; IL-2, 458 bp; IL-4, 301 bp; IL-6, 627 bp; TNF-α, 440 bp; IL-10, 301 bp high incidence of IL-10 mRNA detected. On the other hand, IL-6 and IL-10 mRNA was detected in only one and two of the normal breast tissues respectively.

### MATERIALS AND METHODS

### Patients

Breast tissue samples were obtained from the Department of Histopathology at the Flinders Medical Centre, Adelaide, Australia. Ethics approval was given by the Committee for Clinical Investigation for the use of human tissue in this project. Within an hour of surgical removal, the tissues were embedded in Tissue-Tek (Miles, USA, lot no. 0885053), snap frozen in isopentane cooled by liquid nitrogen. A total of 26 breast tumours were examined for cytokine mRNA expression. Normal breast tissue samples were collected from five patients undergoing breast reduction and from sites remote to the tumour site in six patients undergoing total mastectomy. Histological diagnosis of the tumours was performed separately by a histopathologist. The clinical features of the breast cancer patients are outlined in Table 1 Among the 25 breast tumours examined by immunohistochemistry, 22 were infiltrating ductal carcinomas of different grades and three invasive lobular carcinomas as assessed histologically.

### Cryostat sectioning

Cryostat serial sections from the frozen tissue blocks were cut at a setting of 6 μm at –20°C, fixed on albumin-coated glass slides in ice-cold acetone for 5 min, air dried for 2–3 hours and stored at –20°C. Sections cut at a setting of 20 μm were used in the extraction of total RNA.

### Peripheral blood lymphocyte isolation

A 20-ml aliquot of peripheral blood was collected from a volunteer and diluted 1:1 with phosphate-buffered saline (PBS). The diluted blood was overlayed onto 10 ml of Lymphoprep (Nycomed Pharma AS, Prod No 1001967) and centrifuged at 2000 *g* for 20 min. The interface layer containing mostly lymphocytes was collected, washed twice with PBS and the cell pellet resuspended at $1 \times 10^6$ cells $ml^{-1}$ in RPMI-1640 medium + 10% fetal calf serum

© Cancer Research Campaign 1997

**Table 3** Detection of cytokine mRNA in breast tumours

| Tumour | IL-2 | IL-4 | IL-6 | IL-10 | TNFα |
|---|---|---|---|---|---|
| I7 | – | – | + | + | – |
| I13 | – | – | + | + | + |
| I16 | – | – | + | + | + |
| I19 | – | – | – | – | – |
| I20 | – | ND | + | + | + |
| I21 | – | – | – | – | – |
| I22 | – | – | – | + | + |
| I23 | – | – | – | + | – |
| I24 | – | – | + | + | – |
| I25 | – | – | – | – | – |
| I26 | – | – | – | – | – |
| I27 | – | – | – | – | – |
| I28 | – | – | + | + | + |
| I30 | – | – | + | + | + |
| I31 | – | + | – | + | + |
| I48 | – | – | – | + | – |
| I62 | – | – | + | – | – |
| I64 | – | – | + | + | + |
| I66 | – | + | + | – | – |
| I70 | – | – | + | + | – |
| I72 | ND | – | + | – | ND |
| J17 | – | – | + | + | + |
| J18 | – | – | – | – | – |
| J24 | – | – | – | + | – |
| J33 | ND | – | – | – | – |
| J38 | – | – | – | + | – |

Within an hour of surgical removal, breast tumours were snap frozen in liquid nitrogen-cooled isopentane. Total RNA was isolated from frozen tissue sections, cDNA synthesized and subsequently used in PCR assays with cytokine-specific primer pairs. All tissues showed positive amplification of β-actin mRNA. All tissues showed amplification of CD3δ mRNA, indicating the presence of TILs. Total RNA isolated from PHA-stimulated PBLs was used for a positive control for amplification of cytokine mRNA. The amplified products were electrophoresed on a 2% agarose gel, stained with ethidium bromide and viewed under UV illumination. +, amplified product detected; –, no amplified product detected; ND, not determined.

**Table 4** Detection of cytokine mRNA in normal breast tissues

| Normal | IL-2 | IL-4 | IL-6 | IL-10 | TNF-α |
|---|---|---|---|---|---|
| Nb1 | – | – | – | – | – |
| Nb2 | – | – | – | – | – |
| Nb3 | – | – | – | – | – |
| Nb4 | – | – | – | – | – |
| Nb5 | – | – | + | – | – |
| Nb6 | – | – | – | – | – |
| Nb7 | – | – | – | – | – |
| Nb9 | – | – | – | – | ND |
| Nb10 | – | – | – | + | – |
| Nb11 | – | – | – | + | – |
| Nb12 | – | – | – | – | – |

Within an hour of surgical removal, normal breast tissues were snap frozen in liquid nitrogen-cooled isopentane. Total RNA was isolated from frozen tissue sections, cDNA synthesized and subsequently used in PCR assays with cytokine-specific primer pairs. All tissues showed positive amplification of β-actin mRNA. All tissues showed positive amplification of CD3δ mRNA, indicating the presence of TILs. Total RNA isolated from PHA-stimulated PBLs was used for a positive control for amplification of cytokine mRNA. The amplified products were electrophoressed on a 2% agarose gel, stained with ethidium bromide and viewed under UV illumination. +, amplified product detected; –, no amplified product detected; ND, not determined.

(FCS). The cells were stimulated with phytohaemagglutinin (PHA) at a final concentration of $5\mu$ ml$^{-1}$ for 5 or 24 h in a 37°C incubator with a 5% carbon dioxide atmosphere.

### Total RNA extraction from cellular suspensions

Cytoplasmic RNA was prepared according to (Beckman et al, 1994). Briefly, cellular suspensions were centrifuged for 12 000 ***g*** for 2 min, washed twice in diethylpurocarbonate (DEPC)-treated PBS, pH 7.2, by centrifuging at 6500 ***g*** for 2 min at room temperature. The pellet was resuspended in 100 μl of NP-40 lysis solution (0.025 M sodium chloride, 0.5 M Tris-HCl, pH 7.5, 0.05 M, magnesium chloride, 10% (v/v) NP-40, 200 mM vanyl ribonucleoside complexes [VRCs] (Gibco BRL no. 5522A), vortexed for 10 s and centrifuged at 6500 ***g*** for 1 min to pellet the nuclei. The supernatant was carefully transferred to a new Eppendorf tube to which 300 μl of buffer A (0.08 M sodium acetate, 0.1 M sodium chloride, 0.0002 M EDTA) and 10 μl of 20% sodium dodecyl sulphate (SDS) was added and mixed well. An equal volume of phenol–chloroform–isoamyl alcohol (PCI) was added, mixed well and centrifuged for 5 min at 13 000 ***g*** at room temperature. The upper aqueous phase was collected and the extraction with PCI repeated. In the final extraction, an equal volume of chloroform – isoamyl alcohol (24:1) alone was used to remove any traces of phenol. The upper aqueous layer was stored at –70°C.

### Total RNA extraction from frozen tissue

A range of 3–5 serial sections of frozen breast tissue of 20-μm thickness were cut and placed into 800 μl of ice-cold RNAzol B solution (Biotec Laboratories, cat no. CS-104) and total RNA isolated according to manufacturer's instructions. Briefly, the solution was homogenized for 5 min using a hand pellet mixer. An 80-μl aliquot of chloroform–isoamyl alcohol was added, incubated at 4°C for 5 min and centrifuged for 15 min at 12 000 ***g*** at 4°C. The upper aqueous layer was precipitated with 400 μl of isopropanol for 45 min at 4°C. After centrifuging at 12 000 ***g*** for 15 min at 4°C, the RNA pellet was washed with 75% alchohol, briefly air dried and redissolved in diethyl pyrocarbonate-treated water (0.1% v/v; DEPC, Sigma Chemical, USA, cat no. D-5758).

### RT-PCR assay

First-strand synthesis of cDNA from total RNA extracted from PHA-stimulated peripheral blood lymphocytes (PBLs) or frozen breast tissue as described above was performed at 37°C using 1.5 μl of Moloney murine leukaemia virus reverse transcriptase (200 units ml$^{-1}$; Gibco, cat. no. 8025SB) in the presence of dNTPs at a final concentration of 1.25 mM each (Promega, cat. no. U1240), DTT (Gibco, cat. no. Y00147), RNasin (40 000 units ml$^{-1}$; Promega, cat. no. N2511) and oligo dT (0.8 μg μl$^{-1}$; Promega, cat. no. C110A). RT-PCR assays were performed using cDNA as template in the presence of sense and antisense primer mixes (see Table 2) in final volumes of 50 μl. The sequence of the primers was designed to amplify both introns and exons of a region of the desired gene to ensure amplification of cDNA and not genomic DNA. Amplification of β-actin mRNA was used as a positive control for intact mRNA of any source. Amplification of the CD3δ mRNA was a positive control for the presence of T lymphocytes. In each RT-PCR assay, cDNA synthesized from total RNA extracted from PHA-stimulated peripheral blood lymphocytes was

*© Cancer Research Campaign 1997*

used as positive controls for amplification of cytokine mRNA. In the 36 cycles using a Perkin Elmer thermal cycler, the denaturation step was at 94°C for 60 s, the annealing step at 55°C for 120 s and the final extension step at 74°C for 3 min. Amplified products were electrophoresed in 2% agarose gels, stained with ethidium bromide and photographed under UV transillumination.

### RESULTS

### Cytokine gene transcription

The transcription of the IL-2, IL-4, IL-6, IL-10 and TNF-α genes was examined by RT-PCR in 26 tumours and 11 normal breast tissues. Figure 1 illustrates the amplified products for a representative breast tumour and normal breast tissue. Tables 3 and 4 summarize the detection of cytokine mRNA expression in human breast tumours and normal breast tissues respectively. Amplification of β-actin mRNA was successful in each tissue sample. Each tissue sample showed positive amplification of CD3δ mRNA, indicating the presence of TILs in the total RNA isolated from each tissue. IL-2 mRNA was not detected in any of the tumours. IL-4 mRNA was detected in only 2 of 26 tumours. By contrast, in 13 of 26 tumours, IL-6 mRNA was detected and TNF-α mRNA was detected in 9 of 26 tumours. IL-10 mRNA was detected in 16 of 26 tumours.

In contrast to the pattern of cytokine expression in the tumour tissues, IL-10 mRNA was detected in only two of the normal tissues and IL-6 mRNA was detected in only one of these cases. IL-2, IL-4 and TNF-α mRNA were not detected in any of the normal breast tissues.

### DISCUSSION

In this study, the expression of cytokine mRNA expression in human breast tumours and normal breast tissues was investigated. Heterogeneous cytokine mRNA profiles were observed within the tumours. A consistent feature was the inability to detect IL-2 and IL-4 mRNA in the breast tumours. In contrast, IL-10 mRNA was detected in over 50% of the tumours. Lack of detection of IL-2 and IL-4 mRNA combined with the growth of breast carcinomas suggested a failure of TIL activation.

IL-10 mRNA was detected in more than 50% of the tumours. IL-10 is known to have inhibitory effects on T-cell proliferation and function as well as IL-2 production (De Waal Malefyt et al, 1991*a*, *b*; 1993) Moreover, IL-10 has also been shown to inhibit antigen presentation by macrophages and Langerhans cells (LCs) as well as the presentation of tumour-associated antigens by tumour cells (Ding et al, 1993; Enk et al, 1993; Matsuda et al, 1994; Beissert et al, 1995). The detection of IL-10 mRNA in more than half of the breast tumours and the failure to detect IL-2 mRNA is consistent with previous work shown by others that IL-10 may have an inhibitory role on IL-2 production and T-cell activation. Indeed, lack of detection of IL-2 expression with selective expression of IL-10 in TILs derived from human renal cell carcinoma (RCC) was recently reported in human renal cell carcinomas, further implicating a role for IL-10 in the immunosuppression of TILs (Nakagomi et al, 1995).

Production of IL-10 is associated with the induction of anergy in T lymphocytes. Becker et al (1993) demonstrated the association of IL-10 with the induction of clonal anergy of a human $CD4^+$ T-cell clone by autologous MHC class $II^+$ melanoma. Co-culturing of the melanoma-derived $CD4^+$ T-cell clone with the melanoma cell line derived from the same patient failed to induce T-cell proliferation or IL-2 production. This induction of anergy was accompanied by high amounts of IL-10 mRNA and protein with very little IL-2 produced as detected by ELISA assays and RT-PCR assays (Becker et al, 1994). Co-culturing of the $CD4^+$ T-cell clone with the same autologous melanoma cells transfected with B7 cDNA resulted in IL-2 production, T-cell proliferation as well as significantly lower levels of IL-10 mRNA and protein (Becker et al, 1994). This suggested that IL-10 was important in maintaining anergy in T-cell clones that had been stimulated by autologous MHC class $II^+$ tumour cells in the absence of co-stimulatory molecules. The failure to detect IL-2 mRNA but the detection of IL-10 mRNA in the breast tumours may reflect the cytokine expression of TIL that have been rendered unresponsive or anergic. The source of IL-10 mRNA remains under question in light of recent studies that have demonstrated production of IL-10 by different human carcinoma cell lines (Gastl et al, 1993; Smith et al, 1994). It is possible that the tumour cells themselves may secrete IL-10 to inhibit T-cell function directly or indirectly by inhibiting the presentation of antigen by tumour cells or APCs. In situ hybridization or in situ PCR would confirm the source of IL-10 mRNA in these tumours.

The cytokine mRNA pattern was compared between individual tumours characterized by different clinical parameters, such as local vs metastatic tumours, and infiltrating ductal carcinomas of different grades, to determine if any significant trends would emerge. However, no differences or correlations were observed in any of these comparisons. This suggested that the cytokine expression was characteristic of individual breast tumours and was not directly related to clinical features or the density of lymphocytic infiltration.

In summary, we found that the cytokine mRNA expression in breast tumours was heterogeneous. The striking result of this study was the strong presence of IL-10 mRNA in the majority of tumours and the lack of detection of IL-2 and IL-4 mRNA. These results suggest that whereas TILs may have been previously activated, their anti-tumour activity may be suppressed by the presence of IL-10. Thus, the presence of IL-10 in aggressively growing tumours may reflect an inhibitory role of IL-10 on T-cell function as well as a crucial role in maintaining anergy.

### REFERENCES

Becker JC, Brabletz T, Czerny C, Termeer C and Brocker EB (1993) Tumour escape mechanisms from immunosurveillance: induction of unresponsiveness in a specific MNC-restricted $CD4^+$ human T cell clone by the autologous MHC class $II^+$ melanoma. *Int Immunol* **5**: 1501–1508

Becker JC, Czerny C and Brocker EB (1994) Maintenance of clonal anergy by endogenously produced IL-10. *Int Immunol* **6**: 1605–1612

Beckman I, Sheperd K, Dimopoulos K, Ahern M, Firgaira F and Bradley J (1994) Differential expression and regulation of cytokine mRNAs in normal human CD45R T cell subsets. *Cytokine* **6**: 116–123

Beissert S, Hosoi J, Grabbe S, Asahina A and Granstein R D (1995) IL-10 inhibits tumor antigen presentation by epidermal antigen-presenting cells. *J Immunol* **154**: 1280–1286

Chin Y, Janseens J, Vandepitte J, Vandenbrande J, Opdebeek L and Raus J (1992*a*). Phenotypic analysis of tumor-infiltrating lymphocytes from human breast cancer. *Anticancer Res* **12**: 1463–1466

De Waal Malefyt R, Abrams J, Bennett B, Figdor C G and De Vries JE (1991*a*) Interleukin 10 (IL-10) inhibits cytokine synthesis by human monocytes: an autoregulatory role of IL-10 produced by monocytes. *J Exp Med* **174**: 1209–1220

© Cancer Research Campaign 1997

De Waal Malefyt R, Haanen J, Spits H, Roncarolo MG, Te Velde A, Figdor C, Johnson K, Kastelein R, Yssel H and De Vries JE (1991*b*) Interleukin 10 (IL-10) and viral IL-10 strongly reduce antigen-specific human T cell proliferation by diminishing the antigen-presenting capacity of monocytes via downregulation of class II major histocompatibility complex expression. *J Exp Med* **174**: 915–924

De Waal Malefyt R, Yssel H and De Vries JE (1993) Direct effects of IL-10 on subsets of human CD4+ T cell clones and resting T cells. *J Immunol* **150**: 4754–4765

Ding L, Linsley PS, Huang LY, Germain RN and Shevach E M (1993) IL-10 inhibits macrophage costimulatory activity by selectively inhibiting the up-regulation of B7 expression. *J Immunol* **151**: 1224–1234

Enk AH, Angeloni VL, Udey MC and Katz SI (1993) Inhibition of langerhans cell antigen-presenting function by IL-10. *J Immunol* **151**: 2390–2398

Gastl GA, Abrams JS, Nanus DM, Oosterkamp R, Silver J, Liu F, Chen M, Albino AP and Bander NH (1993) Interleukin-10 production by human carcinoma cell lines and its relationship to interleukin-6 expression. *Int J Cancer* **55**: 96–101

Heo DS, Whiteside TL, Johnson JT, Chen K, Barnes EL and Herberman RB (1987) Long-term interleukin 2-dependent growth and cytotoxic activity of tumor-infiltrating lymphocytes from human squamous cell carcinomas of the head and neck. *Cancer Res* **47**: 6353–6362

Luscher U, Filgueira L, Juretic A, Zuber M, Luscher NJ, Heberer M and Spagnoli GC (1994) The pattern of cytokine gene expression in freshly excised human metastatic melanoma suggests a state of reversible anergy of tumor-infiltrating lymphocytes. *Int J Cancer* **57**: 612–619

Matsuda M, Salazar F, Petersson M, Masucci G, Hansson J, Pisa P, Zhang QJ, Masucci MG and Kiessling R (1994) Interleukin 10 pretreatment protects target cells from tumour- and allo-specific cytotoxic T cells and downregulates HLA class I expression. *J Exp Med* **80**: 2371–2376

Merlo A, Juretic A, Zuber M, Filgueira L, Luscher U, Caetano V, Ulrich J, Gratzl O, Heberer M and Spagnoli GC (1993) Cytokine gene expression in primary brain tumours, metastases and meningiomas suggests specific transcription patterns. *Eur J Cancer* **29A**: 2118–2125

Miescher S, Whiteside TL, Moretta L and Von Fliedner V (1987) Clonal and frequency analyses of tumor-infiltrating T lymphocytes from human solid tumors. *J Immunol* **138**: 4004–4011

Nakagomi H, Pisa P, Pisa EK, Yamamoto Y, Halapi E, Backlin K, Juchlin C and Kiessling R (1995) Lack of interleukin-2 (IL-2) expression and selective expression of IL-10 mRNA in human renal cell carcinoma. *Int J Cancer* **63**: 366–371

Ostenstad B, Lea T, Schlichting E and Harboe M (1994) Human colorectal tumour infiltrating lymphocytes express activation markers and the CD45RO molecule, showing a primed population of lymphocytes in the tumour area. *Gut* **35**: 382–387

Pisa P, Halapi E, Pisa EK, Gerdin E, Hising C, Bucht A, Gerdin B and Kiessling R (1992) Selective expression of interleukin 10, interferon γ and granulocyte-macrophage colony-stimulating factor in ovarian cancer biopsies. *Proc Nat Acad Sci* **89**: 7708–7712

Reilly EB and Antognetti G (1991) Increased tumor-specific CTL activity in human tumor-infiltrating lymphocytes stimulated with autologous tumor lines. *Cell Immunol* **135**: 526–533

Smith DR Kunkel SL Burdick MD, Wilke CA, Orringer MB, Whyte RI and Strieter RM (1994) Production of interleukin-10 by human bronchogenic carcinoma. *Am J Pathol*, **145**: 18–25

Topalian SL and Rosenberg SA (1990) Tumor-infiltrating lymphocytes: Evidence for specific immune reactions against growing cancers in mice and humans. *Imp Adv Oncol* 19–41

Whiteside TL, Miescher S, Hurlimann J, Moretta L and Von Fliedner V (1986) Separation, phenotyping and limiting dilution analysis of T-lymphocytes infiltrating human solid tumors. *Int J Cancer* **37**: 803–811

Whitford P, Mallon EA, George WD and Campbell AM (1990) Flow cytometric analysis of tumour infiltrating lymphocytes in breast cancer. *Br J Cancer* **62**: 971–975

Whitford P, George WD and Campbell AM (1992) Flow cytometric analysis of tumour infiltrating lymphocyte activation and tumour cell MHC Class I and II expression in breast cancer patients. *Cancer Lett* **61**: 157–164

Wimmenauer S, Wintzer H and Von Kleist S (1991) Phenotyping of human tumor-infiltrating lymphocytes before and after exposure to different in vitro stimulation conditions. *Anticancer Res* **11**: 1013–1020

© Cancer Research Campaign 1997


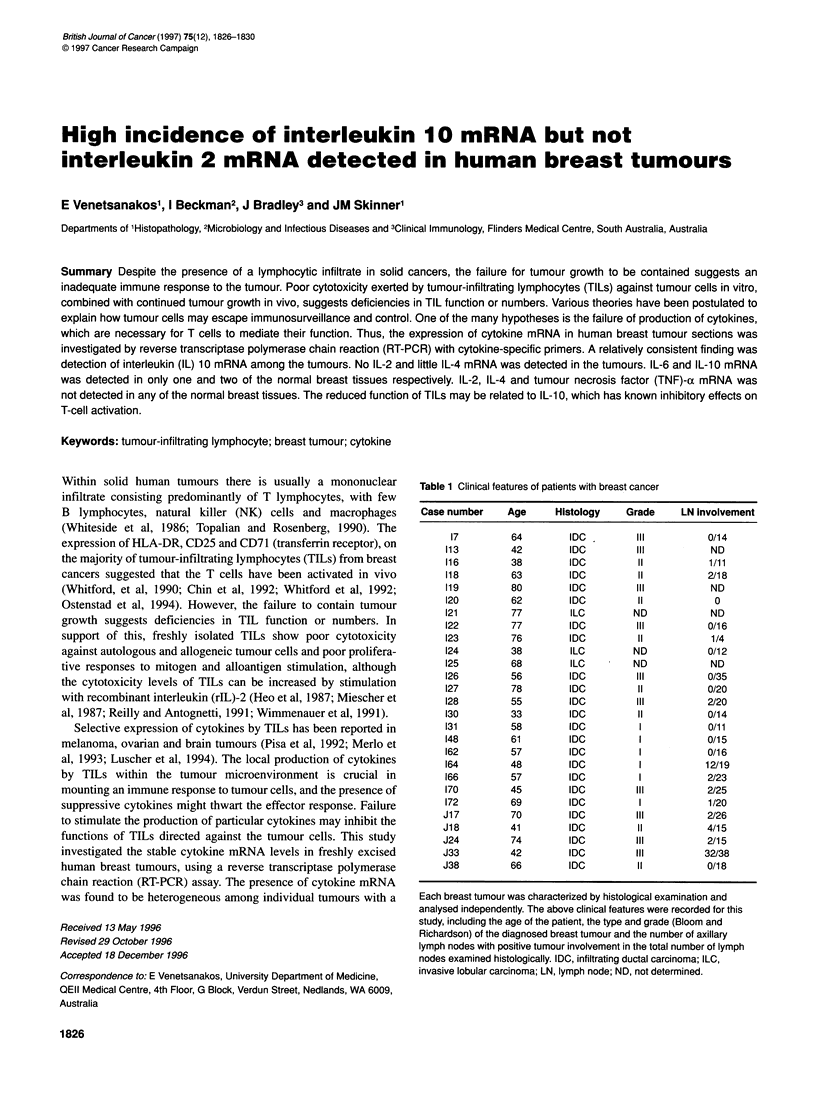

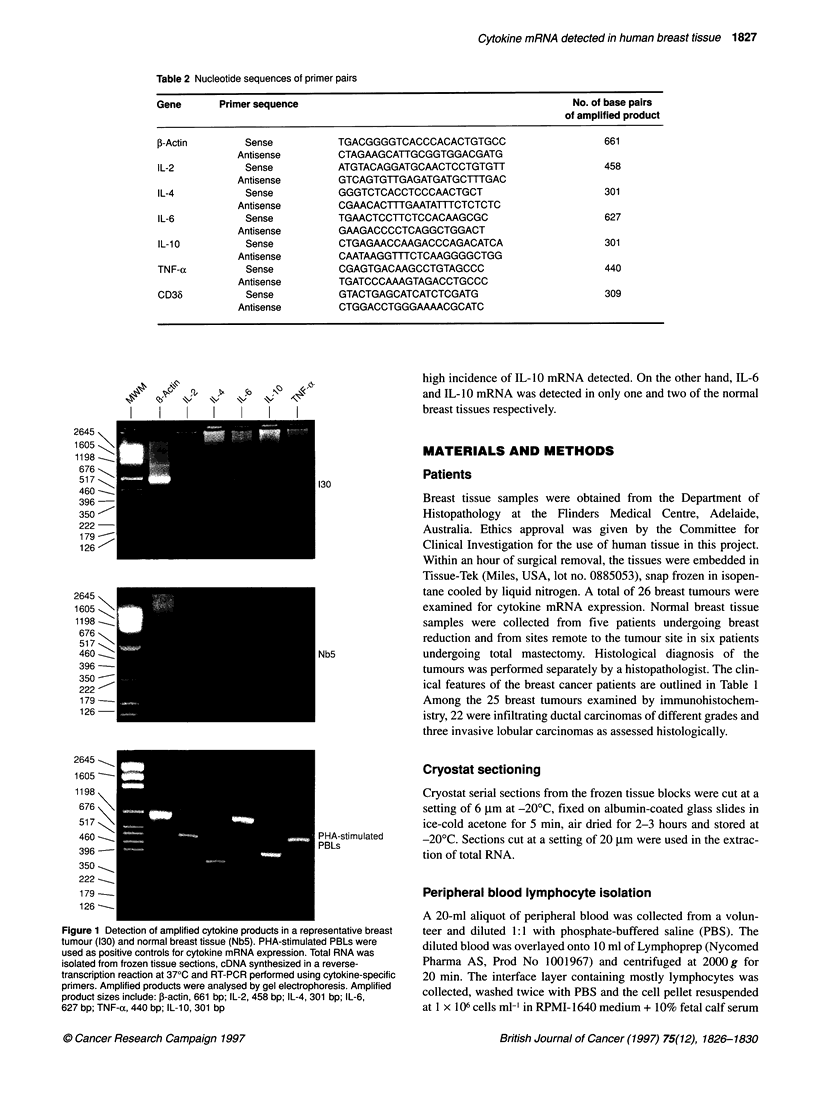

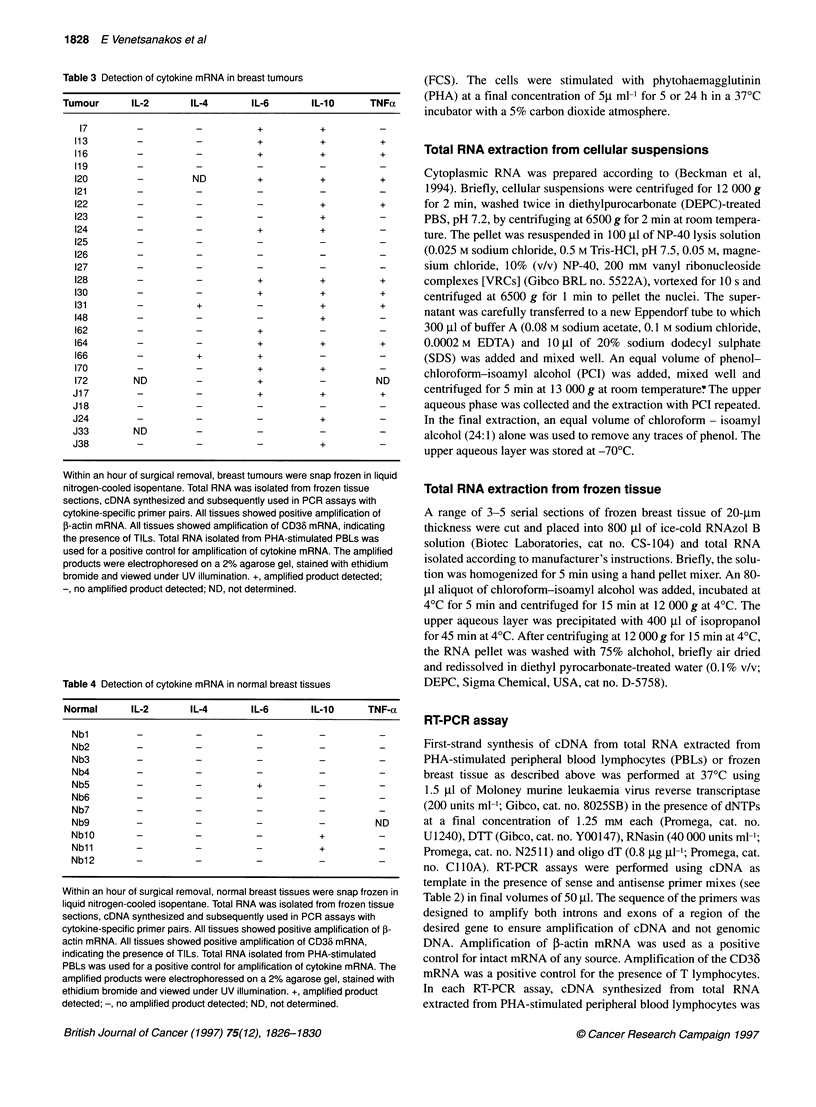

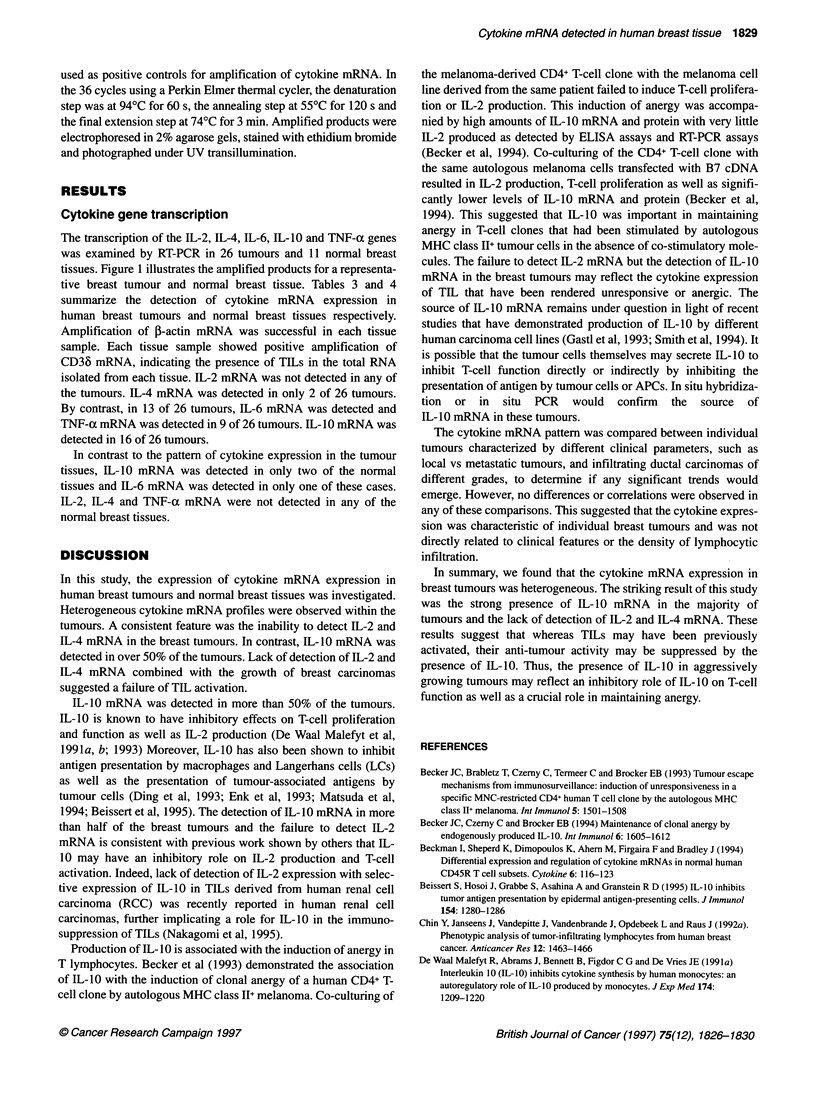

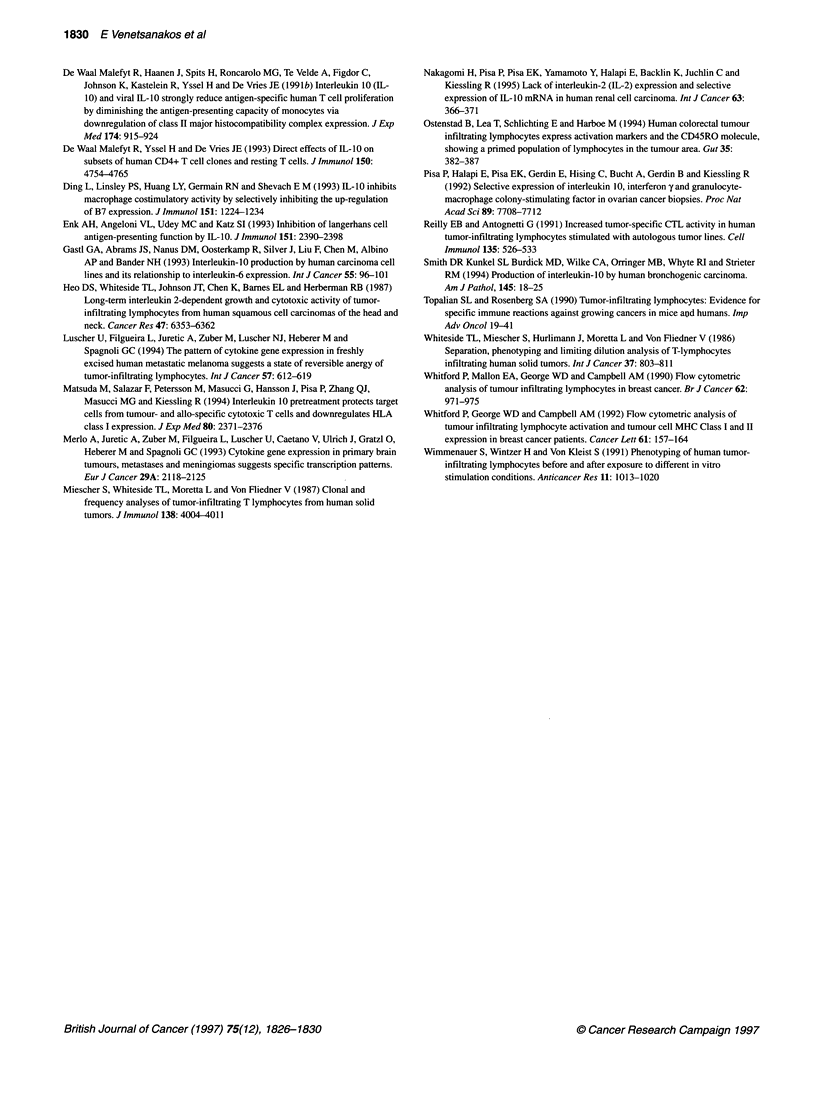


## References

[OCR_00516] Becker J. C., Brabletz T., Czerny C., Termeer C., Bröcker E. B. (1993). Tumor escape mechanisms from immunosurveillance: induction of unresponsiveness in a specific MHC-restricted CD4+ human T cell clone by the autologous MHC class II+ melanoma.. Int Immunol.

[OCR_00522] Becker J. C., Czerny C., Bröcker E. B. (1994). Maintenance of clonal anergy by endogenously produced IL-10.. Int Immunol.

[OCR_00526] Beckman I., Shepherd K., Dimopoulos K., Ahern M., Firgaira F., Bradley J. (1994). Differential expression and regulation of cytokine mRNAs in normal human CD45R T cell subsets.. Cytokine.

[OCR_00531] Beissert S., Hosoi J., Grabbe S., Asahina A., Granstein R. D. (1995). IL-10 inhibits tumor antigen presentation by epidermal antigen-presenting cells.. J Immunol.

[OCR_00536] Chin Y., Janseens J., Vandepitte J., Vandenbrande J., Opdebeek L., Raus J. (1992). Phenotypic analysis of tumor-infiltrating lymphocytes from human breast cancer.. Anticancer Res.

[OCR_00566] Ding L., Linsley P. S., Huang L. Y., Germain R. N., Shevach E. M. (1993). IL-10 inhibits macrophage costimulatory activity by selectively inhibiting the up-regulation of B7 expression.. J Immunol.

[OCR_00571] Enk A. H., Angeloni V. L., Udey M. C., Katz S. I. (1993). Inhibition of Langerhans cell antigen-presenting function by IL-10. A role for IL-10 in induction of tolerance.. J Immunol.

[OCR_00575] Gastl G. A., Abrams J. S., Nanus D. M., Oosterkamp R., Silver J., Liu F., Chen M., Albino A. P., Bander N. H. (1993). Interleukin-10 production by human carcinoma cell lines and its relationship to interleukin-6 expression.. Int J Cancer.

[OCR_00579] Heo D. S., Whiteside T. L., Johnson J. T., Chen K. N., Barnes E. L., Herberman R. B. (1987). Long-term interleukin 2-dependent growth and cytotoxic activity of tumor-infiltrating lymphocytes from human squamous cell carcinomas of the head and neck.. Cancer Res.

[OCR_00586] Lüscher U., Filgueira L., Juretic A., Zuber M., Lüscher N. J., Heberer M., Spagnoli G. C. (1994). The pattern of cytokine gene expression in freshly excised human metastatic melanoma suggests a state of reversible anergy of tumor-infiltrating lymphocytes.. Int J Cancer.

[OCR_00593] Matsuda M., Salazar F., Petersson M., Masucci G., Hansson J., Pisa P., Zhang Q. J., Masucci M. G., Kiessling R. (1994). Interleukin 10 pretreatment protects target cells from tumor- and allo-specific cytotoxic T cells and downregulates HLA class I expression.. J Exp Med.

[OCR_00599] Merlo A., Juretic A., Zuber M., Filgueira L., Lüscher U., Caetano V., Ulrich J., Gratzl O., Heberer M., Spagnoli G. C. (1993). Cytokine gene expression in primary brain tumours, metastases and meningiomas suggests specific transcription patterns.. Eur J Cancer.

[OCR_00605] Miescher S., Whiteside T. L., Moretta L., von Fliedner V. (1987). Clonal and frequency analyses of tumor-infiltrating T lymphocytes from human solid tumors.. J Immunol.

[OCR_00610] Nakagomi H., Pisa P., Pisa E. K., Yamamoto Y., Halapi E., Backlin K., Juhlin C., Kiessling R. (1995). Lack of interleukin-2 (IL-2) expression and selective expression of IL-10 mRNA in human renal cell carcinoma.. Int J Cancer.

[OCR_00617] Ostenstad B., Lea T., Schlichting E., Harboe M. (1994). Human colorectal tumour infiltrating lymphocytes express activation markers and the CD45RO molecule, showing a primed population of lymphocytes in the tumour area.. Gut.

[OCR_00623] Pisa P., Halapi E., Pisa E. K., Gerdin E., Hising C., Bucht A., Gerdin B., Kiessling R. (1992). Selective expression of interleukin 10, interferon gamma, and granulocyte-macrophage colony-stimulating factor in ovarian cancer biopsies.. Proc Natl Acad Sci U S A.

[OCR_00629] Reilly E. B., Antognetti G. (1991). Increased tumor-specific CTL activity in human tumor-infiltrating lymphocytes stimulated with autologous tumor lines.. Cell Immunol.

[OCR_00634] Smith D. R., Kunkel S. L., Burdick M. D., Wilke C. A., Orringer M. B., Whyte R. I., Strieter R. M. (1994). Production of interleukin-10 by human bronchogenic carcinoma.. Am J Pathol.

[OCR_00639] Topalian S. L., Rosenberg S. A. (1990). Tumor-infiltrating lymphocytes: evidence for specific immune reactions against growing cancers in mice and humans.. Important Adv Oncol.

[OCR_00644] Whiteside T. L., Miescher S., Hurlimann J., Moretta L., von Fliedner V. (1986). Separation, phenotyping and limiting dilution analysis of T-lymphocytes infiltrating human solid tumors.. Int J Cancer.

[OCR_00654] Whitford P., George W. D., Campbell A. M. (1992). Flow cytometric analysis of tumour infiltrating lymphocyte activation and tumour cell MHC class I and II expression in breast cancer patients.. Cancer Lett.

[OCR_00649] Whitford P., Mallon E. A., George W. D., Campbell A. M. (1990). Flow cytometric analysis of tumour infiltrating lymphocytes in breast cancer.. Br J Cancer.

[OCR_00659] Wimmenauer S., Wintzer H. O., von Kleist S. (1991). Phenotyping of human tumor-infiltrating lymphocytes before and after exposure to different in vitro stimulation conditions.. Anticancer Res.

[OCR_00541] de Waal Malefyt R., Abrams J., Bennett B., Figdor C. G., de Vries J. E. (1991). Interleukin 10(IL-10) inhibits cytokine synthesis by human monocytes: an autoregulatory role of IL-10 produced by monocytes.. J Exp Med.

[OCR_00551] de Waal Malefyt R., Haanen J., Spits H., Roncarolo M. G., te Velde A., Figdor C., Johnson K., Kastelein R., Yssel H., de Vries J. E. (1991). Interleukin 10 (IL-10) and viral IL-10 strongly reduce antigen-specific human T cell proliferation by diminishing the antigen-presenting capacity of monocytes via downregulation of class II major histocompatibility complex expression.. J Exp Med.

[OCR_00561] de Waal Malefyt R., Yssel H., de Vries J. E. (1993). Direct effects of IL-10 on subsets of human CD4+ T cell clones and resting T cells. Specific inhibition of IL-2 production and proliferation.. J Immunol.

